# Depth perception changes following adaptation to cue-dependent invariants

**DOI:** 10.1038/s41598-025-28193-1

**Published:** 2025-12-30

**Authors:** Francesca Peveri, Federico Barban, Andrea Canessa, Silvio P. Sabatini

**Affiliations:** 1 Department of Informatics, Bioengineering, Robotics and Systems Engineering, University of Genoa, via Opera Pia 11a, 16145 Genoa, Italy; 2 IRCCS Ospedale Policlinico San Martino, Largo Rosanna Benzi 10, 16132 Genoa, Italy

**Keywords:** Depth perception, Visual cue conflict, Slant and tilt, Active perception, Perceptual learning, Cue reweighting, Visual system, Biomedical engineering

## Abstract

How does our perceptual system adapt to new invariants? Can the visual system adapt to non-veridical 3D object properties that remain stable under different transformations? To investigate this, we employed a two-cue depth stimulation paradigm where disparity and texture were in conflict. We found that active visuomotor interaction with a metameric 3D planar surface (i.e., self manipulation of a metameric 3D planar surface) drives adaptation, altering the perceived match of different combinations of stereo and texture information. Notably, this adaptation occurred through action-driven exposure to visual invariants alone, without the need for explicit sensorimotor error feedback. Adopting a 3D vector-sum model to jointly account for both slant and tilt weighting contributions, we analyzed the effects of dynamic and active training on cue integration. By comparing pre- and post-training perceptual judgments, we found that such training induces a post-training cue reweighting, favoring one cue over the other. Control experiments confirmed that this effect requires the dynamic, coherent manipulation of both cues since no significant adaptation occurred when motor action was yoked to a single cue in isolation. Overall, these results demonstrate that new, coherent precepts emerge when sensory cues are dynamically manipulated in a coherent manner, thereby adapting cue-integration mechanisms to new high-level structural invariants. Our work underscores that active exploration is a crucial mechanism for perceptual learning, facilitating the grouping of features defined by systematic (i.e., invariant) relationships among structural properties of the visual signal (in our case the amount of the cue conflict) thus enabling adaptation to novel environmental statistics.

## Introduction

Relative movement between the observer and objects in the world creates specific patterns in the optic array that directly specify 3D scene properties. The aspects of these patterns that remain invariant can distinguish what is stable from what is changing, and how that change is unfolding^1–3^. Consequently, we perceive rigid objects in motion as single unitary entities, and we simultaneously perceive their trajectories and, more generally, the evolution of the statistics of their retinal images over time. Traditionally, experimental evidence of direct perception refers to experiencing invariants of *real* objects or events that undergo transformations caused by explorations. Our main research goal is to investigate whether a mere active manipulation of visuomotor contingencies^4^ of depth cues (in this case, ’texture’ and ’disparity’) can lead our perceptual system to adapt to *artificial* invariants, i.e., to novel and even unnatural object properties, provided that they are invariant to some kind of transformation. In particular, we address the following questions: (i) Is it possible that experiencing invariants in a dynamic (i.e., active) setting, alters and subverts visual perceptual judgment? (ii) Under what conditions does such an adaptation occur? (iii) Could it be possible that we get a strengthening of one cue on the other(s)?

In order to test this hypothesis, we designed a novel “metastimulus“ by combining depth cues in conflict, creating a perceptual experience unfamiliar to the adult system. Conflictual stimuli (i.e., stimuli that contain component information about the same physical property but signal different values) are particularly suited for this purpose. In fact, perceptual integration processes occur (and remain robust) even in confounding circumstances, where the reliability of individual cues is manipulated^5–10^. Normative integration models are based on the assumption that the resulting percept is a weighted linear combination of the available cues. This integration seeks to minimize overall variance, thereby maximizing perceptual reliability^7,9^or optimizing other indices of accuracy and stability indices of perceptual judgment, e.g^11–13^.,. Typically, in static conditions, psychophysical judgments rely on the cues’ relative reliability (typically two), settling in between the actual values associated with the single-cue stimuli.

The notion that the motor system influences visual perception is not new^14–17^, and situations where two percepts compete for awareness, have been frequently used to investigate the effects of action on perception. However, in those studies, the stimulus usually exhibits a motion independent of the observer’s actions^14,18^. Here, we specifically investigate whether experiencing the visual consequences of voluntary movement could promote perceptual changes that affect perceptual judgment, as observed in e.g^19^.,. In their work, Sedda et al. designed a motor task in which the direction and speed of hand movements were continuously displayed as a plaid moving through an aperture. In that context, they introduced the term “self-operated stimuli” to describe situations in which stimulus parameters are actively manipulated by the observer’s movements. Notably, they found that motor training affects subsequent perception, improving the accuracy of internal representations of stimulus geometry. Our results show that single cue invariants (i.e., related to either ’texture’ or ’disparity’) do not yield significant changes in perceptual judgment tasks. Conversely, when the conflict itself (i.e., the conflictual stimulus configuration) is invariant, then we observe a significant adaptation. Specifically, we observed, dynamically, a different efficacy of texture-based with respect to disparity-based invariants, that carried over to a re-weghting of the two cues in static perceptual judgments, afterwards. This finding provides evidence that perceptual processing is directly shaped by dynamic interaction with sensorimotor contingencies, even in the absence of explicit error feedback.

## Methods

### Subjects

Thirty healthy subjects from Genoa University (16 men and 14 women, 21–32 years of age) participated in this study, yet a subject was excluded as he did not complete the training phase properly, possibly due to task misunderstanding. All participants had normal or corrected-to-normal vision and were screened for their visual acuity using Randot® Stereotest (Stereo Optical Co. (2018)). Binocular stereoacuity was considered acceptable if subjects could identify geometric forms and could complete the graded circle test to at least level seven/eight which corresponds to 40/30 seconds of arc from a distance of 40 cm.

The research, approved by the Research Ethics Committee of the University of Genoa, was performed in accordance with all relevant guidelines and regulations, including the Declaration of Helsinki. Each subject, naive to the purpose of the study, signed an informed consent form conforming to these guidelines. Participants were randomly assigned to one of three groups (10 participants per group), each undergoing visuomotor training under different conditions.

### Experimental setup

In a dimly lit room, subjects were comfortably seated on a height-adjustable chair with their forehead laid against a stabilization bar and the chin positioned on a chin rest, at a viewing distance (VD) equal to 50 cm from the monitor, wearing passive circularly polarized glasses, as depicted in Figure 2(**a**). Visual stimulation was presented on a 3D monitor (LG, 1920x1080, 42”, refresh rate 100 Hz), connected to a DELL Alienware PC equipped with NVIDIA GeForce RTX 3090 Graphics card. Visual stimuli and task procedures were designed using the Unity3D graphics engine (Unity Technologies, San Francisco, CA, version 2021.3.21f) and Shaders–specialized scripts, running on the GPU, that control how objects are rendered on the screen and how the surface of a 3D model interacts with light and other visual effects. Two different tools mediated the interaction, depending on the performed task.

During the pre and post-task, the interaction with the visual stimulation was mediated through a handheld Vive Controller. The circular touchpad was used to control stimulus orientation parameters, jointly slant and tilt (*see Experimental procedure*). The OpenXR package and Unity’s Input System plug-in were used to obtain the position of the subject’s touch as a pair of coordinates (x, y) within the range of [−1, 1], where (0, 0) represents the center of the touchpad. Whenever a touch occurred at a radial distance greater than 0.7 units from the center, an action was triggered. For each action, the system (1) computed the angular position of the touch as $\tau = \arctan (y/x)$, (2) used $\tau$ to set the tilt of the surface, and (3) increased or decreased the slant of the surface along the desired tilt direction $\tau$ by a step of $0.1^\circ$ at each frame. For what concerns the visuomotor training phase, interaction was facilitated by a joystick consisting of an arm with a spherical joint and a Vive Tracker 3.0 (Steam VR Tracking V2.0) mounted on top, all supported by a wooden base. The rotations of the tracker were linked to the rotations of the visual stimulus presented. At the beginning of the experiment, the spherical joint was locked in place to keep it vertical. During the interaction phase, the joint was unlocked, allowing for free manipulation. Two infrared laser emitter units (lighthouses), positioned in two corners of the room, tracked the orientation of the joystick. The advantage of using Vive tracking devices is that they are easily integrated in the Unity framework, with their position and orientation automatically tracked and synchronized in the game engine at a sampling frequency of 90 Hz.

### Stimuli

The visual stimuli were presented within a circular aperture that appeared to be behind the monitor, which was positioned 55 cm from the observer. The aperture had a diameter of 35.5 degrees of visual angle, featured a blurred contour, and was displayed on a black background. The stimuli consisted of 3D surface planes that were oriented in space and defined by two visual cues: texture gradient and binocular disparity. The orientation of the 3D surface is characterized by two angular parameters: slant ($\sigma$) and tilt ($\tau$). Slant refers to the degrees of rotation of the surface relative to a frontoparallel reference plane (i.e., perpendicular to the viewer’s line of sight). Tilt is defined as the direction of the projection of the surface’s normal vector onto the reference plane^20–22^. To avoid introducing perspective cues through texture (which would become dominant for judging the orientation of 3D surfaces), we generated textures using a white Voronoi tessellation pattern with black corners. This texture consists of a regular grid of points, randomly jittered in two dimensions, similar to those used in many previous studies on slant-from-texture perception^9,10,23–25^. The stimuli could either be not conflictual in their visual cues – texture (t) and disparity (d) – both indicating the same orientation in space ($\sigma _t = \sigma _d$ and $\tau _t = \tau _d$), or conflictual, indicating that one or both signaled different orientation values($\sigma _t \ne \sigma _d$ and/or $\tau _t \ne \tau _d$).

**Fig. 1:**
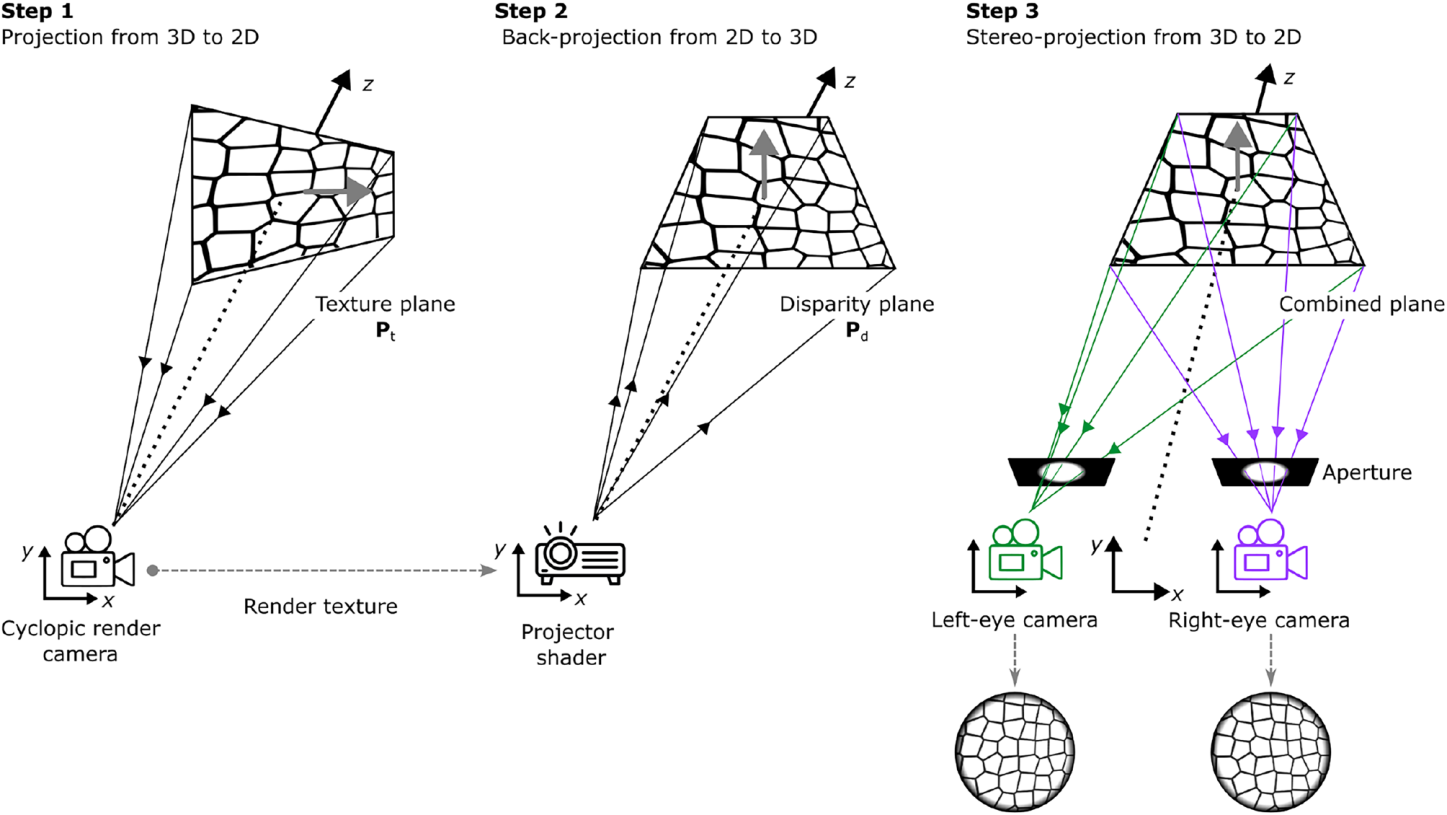
Custom Unity prefab for rendering conflictual 3D stimuli. It consists of two oriented planes (texture plane, $\textbf{P}_t$, and depth plane, $\textbf{P}_d$), which are used to generate images containing information about texture gradients and binocular disparity. A Projector component that leverages shaders, combines these information to produce stereoscopic image pairs. Conflicts between texture and disparity cues arise when the two planes have different orientations, altering the final perceived appearance of the surface. This configuration enables real-time manipulation of $\textbf{P}_t$ and $\textbf{P}_d$, facilitating dynamic interaction with both visual cues. The gray arrow represents the actual normal vector ($\textbf{n}_k$) of the virtual plane in Unity. In the bottom-right corner, two stereoscopic image pairs generated by this pipeline are shown. To perceive the 3D surface orientation, converge your eyes to induce binocular disparity.

We created a custom prefab within the Unity project composed by: (i) two oriented planes (namely the ‘texture plane’ $\textbf{P}_t$ and the ‘depth plane’ $\textbf{P}_d$) to manage the different 3D orientations of the visual cues, whose normal vector is defined as $\textbf{n}_k$ = $\textbf{n}_k$($\sigma$, $\tau$)) = ($\sin \sigma \cos \tau$, $\sin \sigma \sin \tau$, $\cos \tau$), where $k=\{t, d, p\}$, (ii) one cyclopic render camera, (iii) one projector component (implemented through a shader script), and (iv) a pair of stereo virtual cameras. The prefab contained all virtual components and scripts designed and configured to render a conflictual 3D surface following the following steps (see Figure 1):

*Step 1 -* $\textbf{P}_t$ was put at a distance VD in front of the cyclopic camera, at a desired orientation [$\sigma _t$, $\tau _t$]. $\textbf{P}_t$ was texturized with a Voronoi tessellation, generated using custom shader code, at a desired degree of jitter. The cyclopic camera, when viewing $\textbf{P}_t$, rendered its image in a RenderTexture that is passed as input image to a virtual projector.

*Step 2 -* A virtual projector casts an input image onto 3D world’s surfaces within its view frustum, simulating the behavior of a real projector. Our projector shared the same position and orientation as the cyclopic camera. At the same viewing distance as $\textbf{P }_t$, we placed $\textbf{P}_d$ in front of the projector at the desired orientation $[\sigma _d$, $\tau _d]$. As a result, the back-projected image remained consistent regardless of the orientation of the object intersecting the projector’s rays, creating a 3D surface that was physically positioned and oriented as $\textbf{P}_d$, but texturized according to $\textbf{P}_t$.

*Step 3 -* Finally, two stereo cameras, horizontally offset by half of the Interpupillary Distance (IPD) to the left and right of the cyclopic camera, rendered a stereo pair of images–one for each eye. These images create the perception of a stereoscopic textured surface that reflects the physical disparity of the disparity plane. When the defined texture and disparity planes have different slant and tilt orientations, a conflict arises between the two cues, affecting the final surface appearance. This architecture enables independent manipulation of $\textbf{P}_t$ and $\textbf{P}_d$, allowing real-time, dynamic, and independent interaction with the visual cues–binocular disparity and texture.

### Experimental procedure

The experimental procedure was organized into three phases: an initial perceptual judgment task (pre-training phase), a training phase, and a final perceptual judgment task (post-training phase). Subjects were randomly assigned to one of three groups (10 participants per group). Each group underwent a different visuomotor training and consistently completed a corresponding familiarization phase, tailored to the type of training they received.

#### Perceptual judgment task

The purpose of this experimental procedure was to assess participants’ initial perception of the 3D orientation (slant and tilt) of a planar surface in depth, specifically by evaluating the weights assigned to the different visual cues that compose the stimuli. The test employed an adjustment paradigm where participants were required to adjust the 3D orientation of a not conflictual test surface stimulus (by means of the HTC Vive controller’s touchpad) until it matched the perceived orientation of a conflictual fixed reference surface stimulus.

It has generally been observed that, under small conflicts, cue combination follows a weighted averaging approach. In contrast, with larger conflicts, cue combination tends to shift toward cue dominance or ‘cue vetoing’^26^. However, a specific threshold for the transition between combination and dominance cannot be determined, as it varies depending on both the stimulus and the individual subject. Thus, cue discrepancy values were selected on the basis of findings from previous studies in the literature, for which weighted averaging typically occurs^27–29^.

The angular parameters of the reference stimulus were selected from a set of three central slant values ($\sigma _c$ = $15^\circ$, $25^\circ$, and $35^\circ$) and three central tilt values ($\tau _c$ = $0^\circ$, $45^\circ$, and $90^\circ$). Around these values, two conflicting cue configurations were created: [$\Delta \sigma = 30^\circ$, $\Delta \tau = 0^\circ$] and [$\Delta \sigma = 30^\circ$, $\Delta \tau = 45^\circ$]. The conflict was applied such that for the disparity cue, $\sigma _d$ = $\sigma _c$ - $\Delta \sigma / 2$ and $\tau _d$ = $\tau _c$ - $\Delta \tau / 2$, while/similarly for the texture cue, $\sigma _t$ = $\sigma _c$ - $\Delta \sigma / 2$ and $\tau _t$ = $\tau _c$ - $\Delta \tau / 2$.

**Fig. 2:**
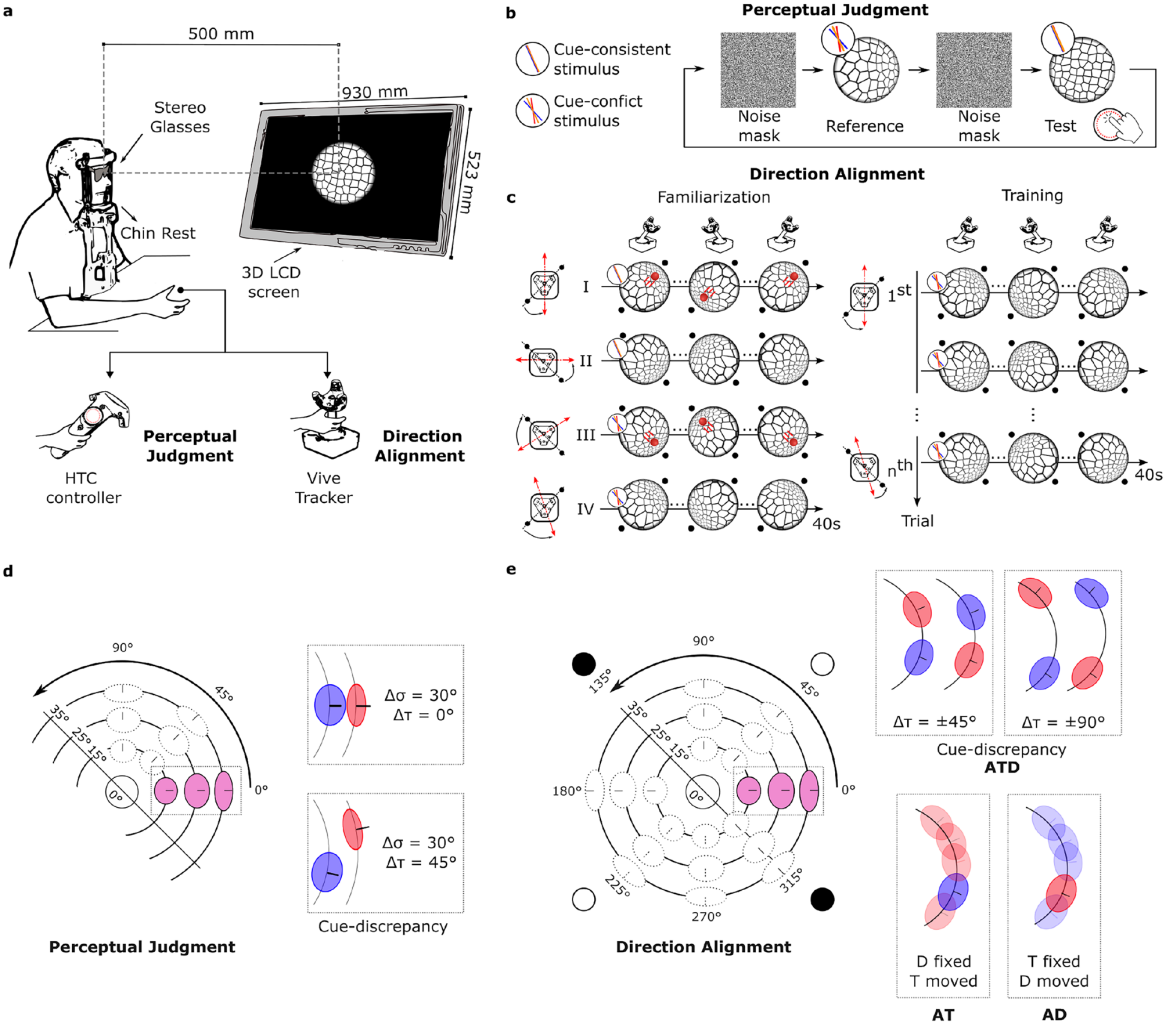
Experimental setup and procedures. (**a**) Stereoscopic setup used in the experiments. During the *Perceptual Judgment Task*, participants interacted with the visual stimuli using the touchpad of the HTC Vive controller, whereas during the *Dynamic Visuomotor Training*, they used a joystick equipped with a Vive Tracker 3.0. Participants sat 50 cm from a 3D monitor, wearing passive circularly polarized glasses, with their head stabilized by a chin rest. (**b**) *Perceptual judgment task* trial timeline. Participants adjusted the 3D orientation of a non-conflictual test surface to match a fixed cue-conflict reference surface. Participants could switch freely between the two views, which were separated by a noise mask. (**c**) Example test trials from the familiarization (left) and training (right) phases. The familiarization phase comprised four 40 s exercises (I–IV) described in detail in the *Familiarization task* section. Visual feedback is indicated schematically as a red ball rolling on the slanted plane in accordance with physical motion. Exercises I–II used a non-conflicting surface with feedback, whereas Exercises III–IV presented a conflicting surface (varied by group) without feedback. The training phase employed the same alignment task but always presenting a conflicting 3D surface providing no visual feedback. (**d**) Representation of the chosen parameters for the *Perceptual Judgment Task*. The combined slant and tilt components form a spherical coordinate system, where lines of latitude represent constant slant and lines of longitude represent constant tilt. Ellipses represent examples of the slant and tilt components of surface orientation for the texture (red) and disparity (blue) cues, respectively, across the three cue-discrepancy conditions built. (**e**) Representation of the chosen parameters for the *Direction Alignment Task*. The ellipses pictorially represent the configurations of the slant and tilt components of surface orientation for the texture (red) and disparity (blue) cues in the joint-control (ATD) training condition and the single-control (AT, AD) conditions. In these last conditions, the semi-transparent disk identifies the orientation of the manipulated cue, while the solid-colored disk represents the orientation of the fixed cue.

As a result, participants were tested in (3 central slants) $\times$ (3 central tilts) $\times$ (2 discrepancy levels) = 18 different conflictual cue configurations, each one repeated five times. Thus, the entire procedure consisted of 90 trials (18 configurations $\times$ 5 repetitions), with the total duration varying depending on the participant, typically ranging between 30 to 40 minutes. A pictorial illustration of the task procedure and the cue configurations tested are presented in Figure 2(**a, d**). For each trial, participants had two different presentations: a reference and a test. For the reference, participants saw a reference stimulus that exhibited one of the conflicts and discrepancies defined above. For the test, participants were presented with an adjustable test stimulus, which consistently displayed congruent binocular disparity and texture information. Participants could adjust the orientation of the test surface to match the perceived orientation of the conflicting reference surface using the controller’s touchpad (as explained in the *Experimental setup* section); conversely, the orientation of the surface in the reference remains fixed. Throughout the trials, participants could freely switch between the reference and the test by pressing the trigger button on the back of the controller as many times as they desired. At each switch, a noise mask was presented to avoid dynamic difference cues. When participants had set the orientation they perceived as equal to the reference surface, they pressed the touchpad button and their decision was saved on a file.

#### Dynamic Visuomotor training

In the training phase, participants performed a *Direction Alignment Task* in which they were instructed to act on the joystick, dynamically controlling the 3D orientation of a conflicting stimulus surface. We specifically designed three distinct training conditions, each differing in the number and type of cues that participants could actively manipulate:

1. *Active Texture-and-Disparity (ATD) Joint-Control Condition:* In this condition, joystick movements simultaneously modified the orientation of both $\textbf{P}_d$ and $\textbf{P}_t$, thus both the visual cues (binocular disparity and texture), while maintaining a fixed discrepancy between them;
2. *Active texture (AT) Single-Control Condition*: In this condition, joystick movements adjusted only the orientation of $\textbf{P}_t$, while $\textbf{P}_d$ remained fixed;
3. *Active disparity (AD) Single-Control Condition*: This condition mirrored the AT condition, but with the role reversed – joystick movements adjusted the orientation of $\textbf{P}_d$, while $\textbf{P}_t$ remained fixed.

Accordingly, we evenly divided participants into three groups of 10, with each group performing only one of the three conditions. The ATD group served as the main test group, while the AT and AD groups acted as control groups. Figure 2(**b, d**) provides a visual representation of the experimental setup and cue configuration. To decouple participants’ hand movements from the movement of the visual stimulus and ensure the task remained focused on visual perception, a random tilt was applied to the manipulated cue planes. In Figure 2(**c**), this is illustrated by a red arrow on the schematic joystick, indicating the direction in which participants had to move so that the resulting visual motion of the stimulus aligned with the direction of the targets (black dots and dashed black line). This prevented the task from becoming purely motor-based and reduced the likelihood of participants developing overly precise hand trajectories by repeating the same movements.

*Familiarization task*- Before starting the training phase, participants completed a familiarization phase designed to introduce them to dynamic visual stimulation and ensure they understood how to properly perform the motor task. The familiarization phase consisted of four exercises - indicated with Romanian letters in Figure 2(**c**) -, which could be repeated at the participant’s discretion, with a minimum of two repetitions. Each exercise lasted 40 s, during which participant should continuously move the joystick as instructed below. In the first pair of exercises, the visual stimulus was always a cue-consistent surface. In the second pair, it was a cue-conflicting surface where the cue(s) (either texture only, or disparity only, or both) controlled by the participant’s movement depended on their assigned group. Moreover, the trials differed also for the presence or not of a visual feedback. In the first and third trials, visual feedback was provided. Participants adjust the 3D orientation of the controlled cue planes (according to their assigned group) to guide a red ball to smoothly rolled back and forth along it, following the direction indicated by two blue targets placed diametrically opposite each other (at $45^\circ$ and $225^\circ$ or at $135^\circ$ and $315^\circ$), positioned outside the stimulus aperture. During the second and fourth trials, no visual feedback was provided. Instead, participants had to perform the same task while mentally visualizing a ball rolling along the surface, following the depth gradient of the stimulus. Notably, no data were collected during the familiarization phase.

*Direction Alignment Task*- Following the familiarization phase, the main training task began. In all conditions, participants were required to make continuous, smooth, back-and-forth movements, ensuring that the perceived tilt of the depth gradient of the conflicting 3D surface remained aligned along the direction indicated by two blue targets placed diametrically opposite each other outside the stimulus aperture. Differently from the familiarization phase, no feedback on performance accuracy was provided. For ATD condition, in each trial both cue planes moved maintaining a fixed tilt discrepancy chosen as $\Delta \tau = \pm 45^\circ$ or $\Delta \tau = \pm 90^\circ$ between them. In AT and AD condition, in each trial only one cue plane moved while maintaining the fixed cue plane at one of three slant values ($\sigma$ = $15^\circ$, $25^\circ$, $35^\circ$) and one of eight tilt values ($\tau$ = $0^\circ$, $45^\circ$, $90^\circ$, $135^\circ$, $180^\circ$, $225^\circ$, $270^\circ$, $315^\circ$). As a result, participants in the ATD group completed 24 trials (4 levels of cue discrepancy $\times$ 2 target positions $\times$ 3 repetitions), while those in the AT and AD control groups completed 48 trials (8 tilt values $\times$ 3 slant values $\times$ 2 target positions). Each trial lasted 40 s and for the entire duration of a trial the system recorded, at 90 Hz sampling frequency, the orientations of both cue planes, $\textbf{P}_t$ and $\textbf{P}_d$.

### Vector sum model for cue weights estimation

Since slant and tilt are angular variables defining 3D orientation within a spherical coordinate system, the most appropriate combination rule for directional cues (e.g., texture and binocular disparity) has been postulated to follow vector summation^30^. Within this framework, we characterize a surface’s 3D orientation using its normal vector. Assuming unbiased perceptual estimates^31^, the mean perceived normal vector, $\mathbf {{n}}_p$, can be modeled as:

1 $$\begin{aligned} \mathbf {{n}}_p = w_t\mathbf {{n}}_t + w_d\mathbf {{n}}_d \end{aligned}$$

where $\textbf{n}_t$ and $\textbf{n}_d$ represent the unit normal vectors, as defined earlier (see *Stimuli* section), associated with texture and disparity cues, respectively ($\Vert \textbf{n}_t \Vert = \Vert \textbf{n}_d \Vert = 1$), and $w_t$ and $w_d$ denote their corresponding weighting coefficients. This system of equations can be solve analytically using the Moore-Penrose pseudo-inverse, as it is overdetermined (three equations for two unknowns: $w_t$ and $w_d$). The overdetermination arises because the vector equation 1 generates three scalar equations (one for each spatial dimension) but only two weighting coefficients to solve for. However, this pseudo-inverse based solution does not inherently guarantee that the resulting combined vector $\textbf{n}_p$ retains unit norm ($\Vert \textbf{n}_p \Vert = 1$), despite $\textbf{n}_t$ and $\textbf{n}_d$ being unit vectors. This limitation stems from the least-squares framework of the psuedo-inverse, which minimized the residual error without enforcing the unit norm constraint. To address this limitation, we reformulate the previous equation incorporating the unit norm constraint. This transfrom the original overdetermined system into a nonlinear constrained optimization problem. The revised system consists of two equations:

$$\begin{aligned} {\left\{ \begin{array}{ll} \Vert w_t\textbf{n}_t + w_d\textbf{n}_d \Vert ^2 - 1 & = 0 \quad \quad \quad \quad (2) \\ \textbf{n}_p \cdot (w_t\textbf{n}_t + w_d\textbf{n}_d) - 1 & = 0 \quad \quad \quad \quad (3) \end{array}\right. } \end{aligned}$$

Expanding these expressions yields a more explicit form that reveals the geometric interpretation of the solution as the intersection between a line and an ellipse in the parameter space ($w_d$,$w_t$) :

$$\begin{aligned} {\left\{ \begin{array}{ll} w_t^2 + w_d^2 + 2 w_t w_d \textbf{n}_d \cdot \textbf{n}_t -1 = 0 \quad \quad \quad \quad (3.1) \\ w_t\textbf{n}_p \cdot \textbf{n}_t + w_d\textbf{n}_p \cdot \textbf{n}_d - 1 = 0 \quad \quad \quad \quad \quad (3.2) \end{array}\right. } \end{aligned}$$

The line parameters are directly determined by the misalignment between between $\textbf{n}_p$ and the individual cues $\textbf{n}_t$ and $\textbf{n}_d$. The ellipse is centered at the origin (0,0), rotated by $-\pi$/4, and with an eccentricity that depends solely on the conflict between the two cues. Notably, a closed-form solution may not always exist: if the perceived normal vector $\textbf{n}_p$ does not lie within the plane defined by $\textbf{n}_t$ and $\textbf{n}_d$, no exact linear combination of the two cues can satisfy both constraints simultaneously. For this reason, we solved the system numerically using the Levenberg–Marquardt algorithm, as implemented in MATLAB’s^32^ lsqnonlin. This method minimizes the residual error between the model’s prediction and the perceptual constraints, yielding the cue weights ($w_d$, $w_t$) that best satisfy the system under both geometric and perceptual constraints.

It is worth noting that the resulting weights ($w_d$, $w_t$) do not sum to one due to their geometrical constraints.

### Statistical analysis

All statistical analyses were performed using R^33^ (version 4.4.1). The dependent variable $w_d$ was first transformed as $2-w_d$ to invert the scale and produce a positively skewed distribution more appropriate for our modelling approach. To account for inter-subject variability and intra-subject correlation, models included random intercepts and random slopes for the relevant predictors, grouped by participant (ID). Two classes of mixed-effects models were employed to answer two distinct questions:

1. **Influence of stimulus parameters on cue weighting.** A single Generalized Linear Mixed Model (GLMM) was fitted to all pre-training data pooled across participants to assess how the central slant ($\sigma _c$) and tilt ($\tau _c$) values influenced the relative weight assigned to the two visual cues. The dependent variable exhibited a logarithmic relationship with the slant parameter that was modulated by tilt, therefore, $\sigma _c$ was log-transformed and entered in interaction with $\tau _c$ to capture this non-linearity. The GLMM was implemented with glmer() function from the *lme4* package^34^ in R and specified as: $w_d \sim \tau _c * \log (\sigma _c) +(1 + \log (\sigma _c) + \tau _c | ID)$, assuming a Gamma error distribution family with $\log$ link function.
2. **Effects of training on cue weighting.** To test how the three different training protocols affected cue weights, data were analysed separately for each experimental group (ATD, AD, AT). Within each group, data were further split by the two tested cue-discrepancy conditions ([$\Delta \sigma = 30^\circ$, $\Delta \tau = 0^\circ$], [$\Delta \sigma = 30^\circ$, $\Delta \tau = 45^\circ$]), yielding to six independent Linear Mixed Models (LMMs) comparing pre- and post-training. LMMs were fitted with lmer() function, using the following general specification: $w_d \sim + \log (\sigma _c)*\tau _c*Time +(1 + \log (\sigma _c) + Time | ID)$.

Model performance and assumptions for both classes of models were evaluated using the performance package^35^, specifically through the check_model() function. When classical LMM assumptions were violated and model performance indicated a better fit for a non-Gaussian family, GLMMs (e.g., Gamma with log link) were preferred. Additional details on model reliability and goodness-of-fit are reported in Supplementary Tables S1–S2 and Supplementary Figures S1–S4.

Finally, post-hoc pairwise comparisons were performed on the estimated marginal means (*emmeans* package^36^), calculated using the emmeans() function, with adjustments for multiple comparisons via Tukey correction.

## Results

To systematically characterize the dynamics of perceptual weighting across the geometric parameter space, we undertook a comprehensive investigation into how slant and tilt modulate visual cue integration. As detailed in the *Methods* section, we obtained the weights associated with texture and disparity cues via a (3D) vector sum that does not depend on the individual reliability of each cue. Consequently, this approach yields a weight that reflects the overall orientation rather than the separate components (slant and/or tilt). For this reason, we then probed more deeply the nature and magnitude of any interaction between these two parameters. Finally, we examined how the perceptual system adapts following dynamic interaction by comparing cue weights before and after training across the three experimental groups (ATD, AT, AD).

### Slant effect on cue weighting

**Fig. 3:**
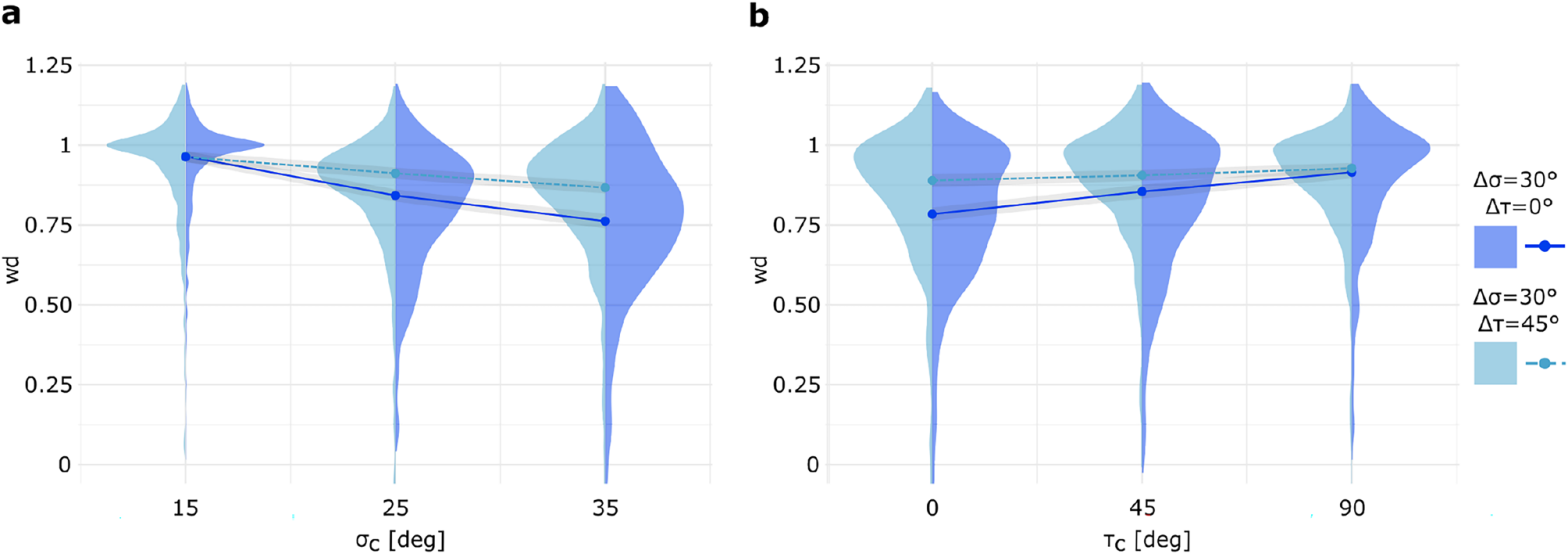
Distribution of w_d_ as a function of (**a**) $\sigma _c$ and (**b**) $\tau _c$ (x-axis) under tested discrepancy conditions (color-coded). Model estimated median values for the discrepancy condition [30$^\circ$, 0$^\circ$] (solid black line) and the [30$^\circ$, 45$^\circ$] condition (dashed black line) from pre-training Perceptual judgment task data (n = 29), are shown with 95% confidence bands (shaded regions), superimposed on half-violin plots representing the observed data distributions. The right half-violin plots (dark gray) correspond to the [30$^\circ$, 0$^\circ$] discrepancy condition, while the left half-violin plots (light gray) represent the [30$^\circ$, 45$^\circ$] discrepancy condition.

In the current study, the [$30^\circ , 0^\circ$] discrepancy condition (to enhance readability, discrepancy conditions will hereafter be abbreviated in square brackets by omitting the $\Delta \sigma$ and $\Delta \tau$ notation) introduce a 30$^\circ$ angular mismatch between disparity and texture slant signals (as depicted in Figure 2(**b**)). The generalized mixed-effects model, used to analyze the pre-training perceptual judgment task data from all participants (n = 29), showed significant modulation of cue weighting by the slant parameter ($\chi$^2^(1) = 67.54, *p*<0.001, Wald’s chi-squared test). Specifically, the relative weight assigned to disparity cue decreased systematically as the conflict center value ($\sigma _c$) increased, as illustrated in Figure 3(**a**) with black solid line. This inverse relationship indicates that observers rely more heavily on texture information for surfaces with steep slants. Our findings align with those of pioneering studies^9,10^ on how texture reliability varies with slant.

The same effect ($\chi$^2^(1) = 28.4705, *p* < 0.001) persists even when a conflict was introduced between the tilt values provided by the two cues (discrepancy condition: [30$^\circ$, 45$^\circ$], black dashed line in Figure 3(**a**)). Here, the down-weighting of the disparity was less pronounced compared to [30$^\circ$, 0$^\circ$] condition, as indicated by the difference between the solid and dashed lines in Figure 3(**a**). Notably, no effect imputable to the presence of conflict was observed for $\sigma _c$ = 15$^\circ$. This likely arises because subjects heavily weighted the disparity cue, which in this configuration signaled a frontoparallel plane ($\sigma _d$ = 0$^\circ$). Since tilt is undefined for a frontoparallel plane, this introduces a singularity in the parameter space. Moreover, during the experiment, subjects adjusted the overall orientation of the surface stimulus rather than acting on the two parameters independently.

### Tilt effect on cue weighting

Our investigation revealed that the tilt parameter $\tau _c$ exhibits an opposing effect to the slant parameter: for any fixed $\sigma _c$, an increase in $\tau _c$ resulted in a corresponding increase in the disparity weight, as shown in Figure 3(**b**) with black solid line. This relationship was robustly captured by our statistical model ($\chi$^2^(1) = 59.54, *p* < 0.001). Hence the orientation indicated by the disparity cue was consistently closer to frontoparallel than that indicated by the texture (as specified in the *Perceptual judgment task* section), these results suggest that binocular disparity cue becomes more informative when surfaces are slanted at 90$^\circ$ (i.e., rotated away from the observer around the horizontal axis) or more broadly, indicates that tilt affects slant perception. Consistent with this, Higashiyama et al.^37^ found that ground patterns are judged as less slanted than ceiling or side-wall patterns. Furthermore, the preference for the orientation indicated by binocular disparity information in our study may also be linked to the fact that our visual system is optimized for retinal correspondences that resemble natural statistics^38^. Therefore, in our case, top-down inclined disparity might prove to be more ecologically relevant or reliable because they favor retinal correspondences compared to disparities indicating vertical or oblique tilts (i.e., $\tau _c$ = 0$^\circ$ and 45$^\circ$). Given that the disparity cue inherently signals a lower magnitude of slant in our tested configurations, our findings suggest that tilt compromises the reliability of texture cues along the conflict axis, an effect referred to in the literature as ’slant anisotropy’^39^.

Introducing an angular conflict between the tilt signaled by texture and disparity (black dashed line in Figure 3(**b**)) preserved the same trend seen in the [30$^\circ$, 0$^\circ$] discrepancy condition, although the effect size was reduced ($\chi$^2^(1) = 7.86, *p* = 0.005). Moreover, although disparity cues retained a significantly greater overall weighting in perceptual integration compared to other tested cues, incorporating a $\Delta \tau$ across all slant orientations enhanced the stability of disparity-driven contributions, yielding increased consistency across the examined parameter space.

### Modulation of visual cue weighting by the interaction of slant and tilt parameters

**Fig. 4:**
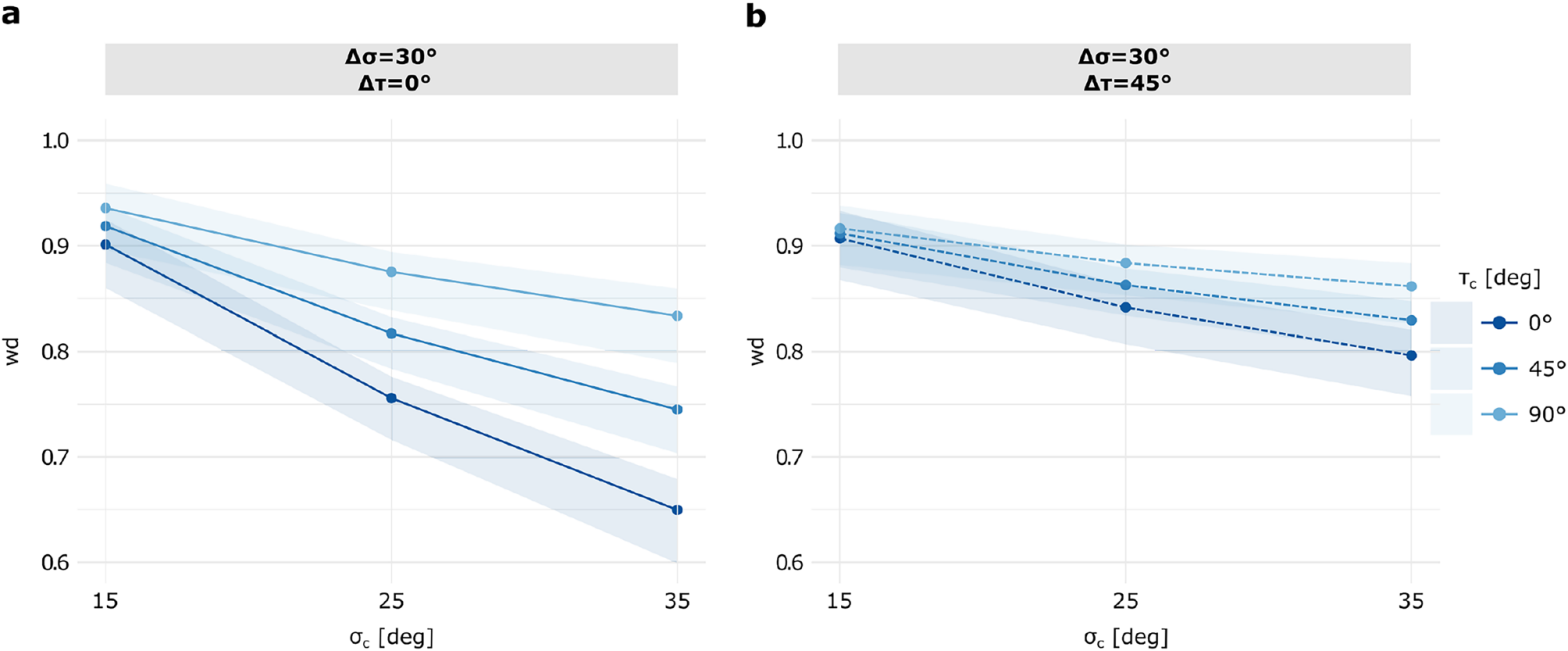
Model estimated marginal means of $w_d$ as a function of $\sigma _c$ (x-axis) and $\tau _c$ (color-coded) under tested discrepancy conditions. (**a**) [30$^\circ$, 0$^\circ$] condition and (**b**) [30$^\circ$, 45$^\circ$] condition, with 95% confidence bands (shaded regions). Disparity cue weights ($w_d$), from pre-training Perceptual Judgment task data are shown for three $\tau _c$ values (0$^\circ$, 45$^\circ$, 90$^\circ$), encoded by a dark-to-light blue color gradient.

Based on our previously presented results, where we highlighted that slant and tilt differently modulate how subjects rely on and therefore integrate depth visual cues. Consequently, we examined in greater/more detail how the weights behave across the parameter space to provide a comprehensive characterization to justify our use of a cue-combination method that takes into account both geometric parameters – which we hypothesize to be intrinsically linked. Figure 4 illustrates the trend of $w_d$ as a function of $\sigma _c$ while varying $\tau _c$ in the condition of (**a**) conflict only on slant ([30$^\circ$, 0$^\circ$]) and (**b**) conflict on both geometric parameters ([30$^\circ$, 45$^\circ$]). There is an interaction between the two geometrical parameters since in the [30$^\circ$, 0$^\circ$] cue-discrepancy, we identified statistically significant differences in perceived slant, and consequently in $w_d$, across all tested $\tau _c$ (*p* < 0.001), as well as tilts. Specifically, for surfaces at both $\sigma _c$ = 25$^\circ$ and $\sigma _c$ = 35$^\circ$, a different tilt elicited a significant perceptual difference ($\tau _c$ = 0$^\circ$ vs 45$^\circ$, 0$^\circ$ vs 90$^\circ$, and 45$^\circ$ $^\circ$ vs 90$^\circ$, *p* < 0.001). In contrast, under the [30$^\circ$, 45$^\circ$] cue-discrepancy condition - where the two visual cues indicate also conflicting tilts - the modulatory effect of slant on cues weighting persisted only when the conflict was centered at $\tau _c$ = 0$^\circ$ and $\tau _c$ = 45$^\circ$ (*p* < 0.001), but not at $\tau _c$ = 90$^\circ$, where weighting remained (quite) uniform across the domain. As noted earlier, in this condition, surfaces/planes with varying tilts did not significantly influence the relative weighting of the two cues. The introduction of a $\Delta \tau$ produced an upward shift in all regression lines (cf. Figure 4(**a**) vs 4(**b**)), indicating an overall increase in the contribution of disparity cue. Table 1 presents the post-hoc pairwise comparison results across all cue-discrepancy conditions, confirming our hypothesis that a perceptual anisotropy exists when texture and disparity cues conflict.

**Table 1:** Table showing the statistical results of GLM-based post-hoc pairwise comparisons (Tukey’s correction) for the cue-weighting analysis from pre-training *Perceptual Judgment* data of all participants (n = 29) across (top) [30$^\circ$, 0$^\circ$] and (bottom) [30$^\circ$, 45$^\circ$] cue-discrepancy. The table lists combinations of $\sigma _c$ and $\tau _c$ parameters, along with the corresponding contrast, effect, standard error (SE), z-ratio, and p-value for each comparison. Significant differences are indicated by p-values less than 0.05. The dot symbol $(\cdot )$ in the tables serves as a placeholder to indicate parameter constancy in specific comparisons. This notation convention helps quickly identify which parameters were actively compared in the contrast (explicit values shown) and which held constant during the analysis (indicated by $\cdot$). For example, the first row in the [30$^\circ$, 0$^\circ$] discrepancy table indicates a comparison between $\sigma _c$ values (15$^\circ$ vs 25$^\circ$) with $\tau _c$ held constant at 0$^\circ$.

$\sigma _c$ [deg]	$\tau _c$ [deg]	contrast	effect	SE	z-ratio	p-value
[$\Delta \sigma$ = 30$^\circ$, $\Delta \tau$ =0$^\circ$]						
.	0	15/25	0.88	0.01	−8.22	<0.001
.	0	15/35	0.81	0.02	−8.22	<0.001
.	0	25/35	0.92	0.01	−8.22	<0.001
25	.	0/45	1.05	0.01	5.43	<0.001
25	.	0/90	1.11	0.02	5.43	<0.001
35	.	0/45	1.08	0.01	7.09	<0.001
35	.	0/90	1.16	0.02	7.09	<0.001
.	45	15/25	0.91	0.01	−6.45	<0.001
.	45	15/35	0.86	0.02	−6.45	<0.001
.	45	25/35	0.94	0.01	−6.45	<0.001
25	.	45/90	1.05	0.01	5.43	<0.001
35	.	45/90	1.08	0.01	7.09	<0.001
.	90	15/25	0.95	0.01	−3.67	0.01
.	90	15/35	0.91	0.02	−3.67	0.01
.	90	25/35	0.96	0.01	−3.67	0.01
[$\Delta \sigma$ = **30**$^\circ$, $\Delta \tau$ = **45**$^\circ$]						
.	0	15/25	0.94	0.01	−5.34	<0.001
.	0	15/35	0.91	0.02	−5.34	<0.001
.	0	25/35	0.96	0.01	−5.34	<0.001
.	45	15/25	0.96	0.01	−4.58	<0.001
.	45	15/35	0.93	0.01	−4.58	<0.001
.	45	25/35	0.97	0.01	−4.58	<0.001

### Post-adaptation cue re-weighting following experience of joint visual invariants

**Fig. 5:**
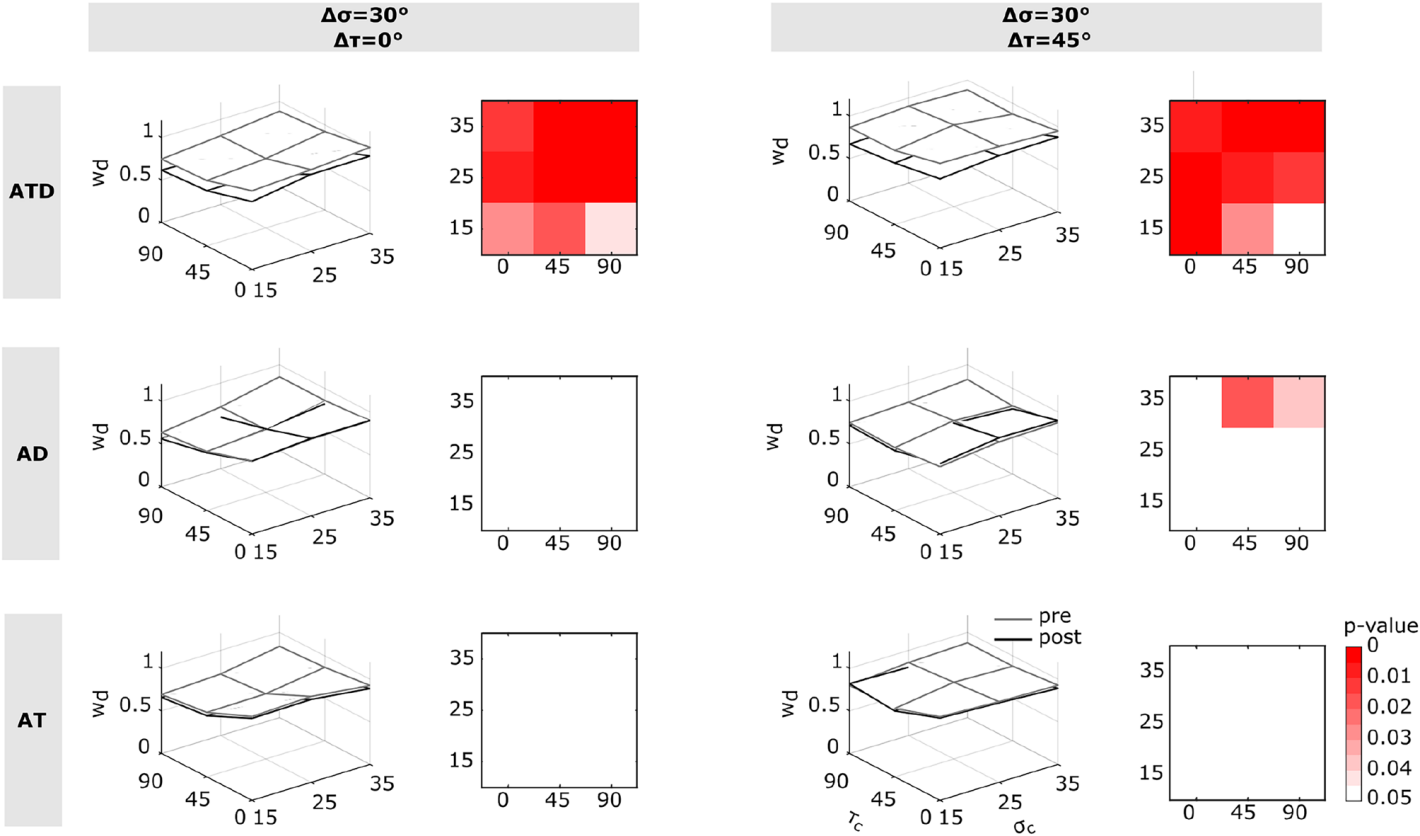
Cue re-weighting after visuomotor training for the three experimental groups. From top to bottom, the 3D representations of pre- and post-training cue weights for the ATD, AD, and AT groups are shown in the ($\sigma _c$, $\tau _c$) parameter domain. The plane illustrates the changes in cue weighting, with the pre-training weights shown in grey and post-training weights in black. The shape of the plane varies according to the cue-discrepancy conditions, reflecting differences in slants and tilt. The second and fourth columns display heatmaps illustrating the statistical significance of the differences found in LM post-hoc pairwise comparisons across combinations of parameter values. The $3\times 3$ matrix represents the combinations of $\sigma _c$ and $\tau _c$ parameters, with a chromatic scale that encodes statistical significance levels, progressing from white (non-significant, *p* $\ge$ 0.05) to red (*p* < 0.01), via increasingly saturated red hues. The *p*-values for each case are reported in tabular form in the Supplementary notes (see Table S2).

To investigate whether and under which conditions experiencing invariants could (consistently) alter perceptual judgments, we compared cue weighting before and after the different dynamic visuomotor training considered. Given that the previous analysis pointed out the non-constant weighting of visual cues – precluding the definition of a unique weight – we assessed variations across all proposed configurations. Figure 5 illustrates how $w_d$’s vary for the pairs of $(\sigma _c, \tau _c)$ in the parameter space, by displaying the results before and after training for the three conflict configurations (columns) and experimental groups (rows). The second and fourth columns in the Figure illustrate $3\times 3$ matrices identifying cue configurations where training elicited statistically significant differences (*p* < 0.05). The ATD group, trained under the joint control of disparity and texture changes, demonstrated a significant reweighting toward the texture cue, in both conditions of discrepancy and for all cue configurations. Unlike the ATD group, which exhibited coherent reweighting, the control groups (AT and AD), which underwent a training for which single cue changes were actively controlled, failed to reflect, as shown by the white matrices in Figure 5, a consistent shift in cue weighting between pre- and post-training, except for some spurious effects observed in the AD group under conditions $\sigma _c$ = 35$^\circ$ at $\tau _c$ = 45$^\circ$ and 90$^\circ$ (*p* = 0.02 and *p* = 0.04). Table S3 in Supplementary reports all the effects from the post-hoc analysis.

## Discussion and conclusions

An adult brain is capable of optimally integrating monocular and binocular cues to estimate 3D spatial orientation in depth^7,9,10^. Pictorial cues – such as texture gradients, linear perspective, and shading – provide critical depth information in static images, while binocular disparity and motion-based signals, like motion parallax, convey dynamic, complementary data. Since individual cues are inherently noisy and often ambiguous^40^, the visual system combines them through a process of weighted integration, where each cue’s influence depends on its context-specific reliability. For example, binocular disparity is highly informative at short viewing distances, whereas information provided by the texture gradients depends critically on surface slant^9^. This cue integration yields more robust and precise estimates of 3D structure, which underpin stable perception and guide goal-directed action. Precisely because of this, stimuli that are ambiguous or contain cue conflicts are excellent candidates to probe both the dynamics of cue weighting and the specific modulatory influence of self-generated movements on perceptual inference. In the present study, perceptual judgments of the 3D orientation of a planar surface were conducted before and after a motor training phase, during which subjects could adapt to different stimulation conditions. To avoid introducing cues other than texture and binocular disparity, stimuli were composed of planar surfaces with a Voronoi tassellation pattern and viewed through a frontoparallel circular aperture, thus removing perspective information. Our study confirmed previous experimental evidence on slant perception that employs perturbation-based paradigms^9,37,41^. More importantly, our results showed that a prolonged interactive experience of action-based (i.e. self-operated) stimulus’ changes leads to a perceptual re-weighting of the single cues, and thus an alteration of perception of static conflictual stimuli presented afterwards. We conclude that dynamic interaction can shape perception in the absence of explicit sensorimotor feedback. Moreover, the observed adaptation strictly depends on the fact that the relative conflict between the to cues remains invariant under transformation (i.e., both cues equally change contingently with action - ATD condition). Control experiments (AT and AD) in which subjects actively manipulate either stimulus’ texture or disparity, while the other remains fixed, showed no significant effect. While these null findings should be interpreted with caution due to the limited sample sizes, which raise the possibility of type II errors, they nonetheless suggest that adaptation is primarily driven by the perceived stimuli (i.e., integrated depth metamer) rather than the individual cues. Interestingly, the re-weighting shifts in favour of texture, suggesting that during motor training, the perception of the rotation of the planar surface (i.e., the time-varying slant) around a transversal axis (i.e., along a tilt direction) is dominated by changes in the monocular retinal image. This agrees with previous evidences that a variation in texture gradient is processed faster than a variation of disparity^16^ when adjustments of ongoing movements to contingent/transient changes in slant are investigated. How this difference in latency affects optimal cue combination dynamically, and how it implies a different recruitment of early vision channels is still an open issue, and it will be specifically addressed in a future work.

It is worth noting that, unlike previous literature^22,25,42^– which treated the two components of surface orientation separately and thereby suggested their independence – our framework treats slant and tilt as integrated quantities rather than independent components. We mathematically formalized the cues using the surface normal vector, therefore rather than assigning separated weights to all the geometrical parameters, we weight the “full” orientation of the cues. While this 3D approach provides a more physically consistent representation of surface orientation in spatial domain, it has inherent limitations. Specifically, this proposed model operates within a purely geometric framework and therefore does not incorporate principles of “optimal cue integration” such as those formalized by maximum-likelihood estimation (MLE). In contrast to Bayesian model of sensory perception^43^, which posits that cues are combined in a statistically optimal manner to maximize the precision of perceptual estimates, here we not account for the reliability of individual cues. MLE posit that, the optimal integration strategy involves computing a weighted average of sensory cues, where the weights are inversely proportional to the uncertainty (or variance) associated with each cue. This process yield to a final estimate that is statistically more reliable than any of the cues alone. Notwithstanding, our results draw attention to the fact that slant and tilt should instead be considered jointly to capture the key effects arising from their interdependence, further justifying the need for a combination method that accounts for both. In future work, we will address this limitation by integrating the geometric and Bayesian approaches to better model perceptual decision-making under uncertainty.

### Action-perception transfer

The way perception influences movements through sensory perturbations is well documented, for example in contexts such as adaptation to force fields or to visuomotor rotation induced by prisms; on the contrary, the mechanisms by which movements affect perception are less known, for example by guiding a persistent improvement of the perceptual discrimination and/or detection capabilities following motor practice with a perceptive stimulus. The effect of self-generated actions on perceptual judgment was investigated by Beats et al.^14,18^ using an ambiguous rotating cylinder composed of clustered dots. The stimulus exhibited motion independently of the observer’s actions. Participants were instructed to match their velocity with that of the rotating cylinder by either pressing a key on a keyboard or manipulating a handle. Two conditions of interest were (1) the motor instructed blocks, where observers rotated the manipulandum clockwise (CW) or counterclockwise (CCW) regardless of the percept, and (2) the motor report blocks, where the manipulandum was rotated based on the current perceptual interpretation of the visual stimulus. Perceptual stability was affected only when perception and movement were congruently related, while incoherent movements did not yield similar effects. These findings demonstrate that actions influence perception only when they are related.

More specifically related to the goal of this study, it has been observed that training can dynamically modify visual cue weighting, particularly when involving conflicting stimuli and haptic feedback aligned with one of the cues. This adaptation demonstrates that humans can behaviorally adjust to changes in the mapping between the environment and sensory signals^44–46^, underscoring the role of sensory plasticity. Ernst et al.^7^ investigated how haptic feedback can alter subsequent visual percepts by changing the weights assigned to different sources of visual information in the presence of inconsistent signals. The authors tested three training conditions: (1) the texture-feedback condition, where haptic feedback was consistent with the slant specified by the texture gradient; (2) the disparity-feedback condition, where haptic feedback aligned with the slant specified by disparity; and (3) a control condition with no haptic feedback. They examined differences between pre- and post-test judgments to assess the impact of the haptic feedback provided during the training phase. The results indicated that when subjects received haptic stimulation consistent with one of the cue-specified slants of a visual stimulus, their subsequent visual percepts aligned more closely with the reinforced cue than they had before training. Using a similar approach, van Beers et al.^47^ required subjects to place an object on a slanted surface where both monocular and binocular cues were provided, which could be inconsistent with one another. They found that the weight of the cue consistent with the haptic feedback increased after the training phase compared to before. Recently, a series of studies^29,48,49^ have explored when and how sensory feedback from visually guided movements improves the accuracy of 3D shape perception. Specifically, these studies focused on the mechanism known as “perceptual cue-reweighting”, a feedback-driven learning process that modifies the relative influences of different sources of 3D shape information in perceptual judgments and motor planning. Using a grasping task, they investigated the occurrence of the cue-reweighting mechanism, hypothesizing that a constant bias in 3D shape perception, could be rapidly resolved through a sensorimotor adaptation mechanism without actual perceptual changes. Conversely, if subjects experience variable, trial-by-trial errors, the only way the motor system can compensate them is by altering its reliance on available visual cues. This adjustment can only occur through sensory feedback, which the motor system uses as training signals. There, again, three distinct phases, a pre- and post-test designed as a metamer matching perceptual judgment task, along with a grasping task for training, were employed to investigate the effect of sensory feedback on reinforcing a specific visual cue of the stimulus. The metamer matching task involved repeatedly presenting subjects with a 30 mm depth cue-consistent stimulus, followed by an adjustable cue-conflict stimulus that participants could incrementally adjust in stereo depth until they perceived a match. In the grasping task, participants reached toward depth-conflicting stimuli under two feedback conditions: (1) haptic for texture, where sensory feedback reinforced the texture cue, and (2) haptic for disparity, where binocular disparity was the reinforced cue. The results from the comparison of pre- and post-training cue weights suggest that persistent movement errors serve as a significant driving signal for the cue-reweighting mechanism. Consequently, an altered correlation between visual and haptic stimulation induces a reweighting toward the reinforced cue. This topic has also been explored to develop perceptual and motor training aimed at improving functional vision in individuals with visual impairments (e.g., strabismus, stereoblindness, amblyopia). Vedamurthy et al.^50^ found that a natural visuomotor task involving depth cues in both consistent and conflictual configurations enabled adults with long-standing deficits in stereo vision to upweight their reliance on stereoscopic cues and enhance their accuracy in detecting slants in depth.

In summary, all these lines of evidence address the role of movement errors as the driving force behind perceptual reweighting, in a “ballistic” setting where errors are driven by a priori perceptual judgment. As in Sedda et al.^19^, here we present a different perspective on the complex relationships between movement, motor learning, and visual perceptual learning. Our findings are consistent with the idea that sensory-prediction errors (i.e., the same types of feedback signals that drive other forms of motor learning) are primarily involved in perceptual changes. However, a key difference with previous studies lies in the fact that the role of feedback in perceptual learning was investigated through static procedure, only. Indeed, [Cesanek-Domini, Hillis-Banks,...] assessed prediction errors as mere sensory feedback (cf. feed-forward or “open-loop” models) through cross-modal comparison of haptic information and single cue estimates of 3D shape, without on-line motor control. Therefore, the authors refer to them as a conflict between actual sensory feedback and an internal prediction of that feedback formed on the basis of *prior* information. Conversely, in our experiment, a continuous visual feedback is used in “closed-loop” to correct the actual course of movement, when controlling the movement of the perceived (virtual) plane during training. The continuous movement intrinsically leads the subject to experience a variable prediction error that cannot be resolved by simple sensorimotor adaptation. Specifically, given the high variability of the cue weights revealed in the pre-training characterization and, the fact that visual surface orientation was continuously manipulated during training, our paradigm precluded simple adaptation. Other feedback signals, such as the proprioceptive one was disrupted by introducing, at each training series, a random tilt in joystick-controlled surface motion, effectively decoupling hand movement from visual surface motion. Notably, our results also demonstrate the role of the persistence of the conditions for the error. Such element of persistence, pivotal for producing visual perceptual changes, can be associated with the invariance of the cues’ conflict in our dynamic experimental setting. Therefore, the present study demonstrates that dynamic interaction and experience of regularities in the sensorimotor flow or in sensorimotor contingencies can influence perception – evidenced by cue-weighting changes in the ATD group – even in the absence of an explicit feedback, previously considered necessary to drive such reweighting.

### Learning invariants under transformations

The idea of an active organism exploring the invariants of an object or event undergoing transformations due to exploration can be traced back to^51–53^ with modern interpretations by^54,55^. However, these works primarily emphasize displacements or transformations, rather than invariants themselves. Gibson’s unique contribution^1^lies in his focus on invariants as revealed through transformations. According to Gibson’s direct perception, visual stimulation is conceptualized as a dynamic flow resulting from relative changes between the environment and the perceiving agent. This dynamic flow exhibits considerable variability; however, certain characteristics remain constant, and emerge as invariants of the optic array, contrasting with the notion of constructing an explicit three-dimensional model (cf^56^.). Importantly, Gibson’s invariants are second-order properties, meaning the invariance in an optic array pertains to the relationship between two varying components–while the components may change, their relational structure remains fixed. Sensorimotor approaches to perception emphasize how sensory information directly informs behavior, aligning with direct theories that elude explicit three-dimensional representations^4^.

For Gibson, the reliability of perception is grounded in the information accessible to the perceiver. Traditional variables from sensation and psychophysics were deemed insufficiently reliable, leading to the identification of higher-order variables as carriers of information. Initially recognized as gradients^2^, these evolved into invariants^57^ and later complexes of invariants^1^. Gradients represent unidimensional measures reflecting orientation changes of objects or textures, while invariants are measures that remain unchanged regardless of viewpoint or interaction. From this perspective, the percepts associated with the different visual stimulations experienced by the subjects of our study during adaptation can be considered as different types of metastimuli depending on the dynamic coherence of the cues. The ATD stimulus may be interpreted as a single stimulus that emerges from a combination of simpler sensory inputs, resulting in a complex perceptual experience. Conversely, control AT and AD stimuli may suggest a scenario where one stimulus (e.g., a static depth cue) modulates or influences the perception of other stimuli (e.g., a moving depth cue). In this sense, the metastimulus is not directly perceived but serves as a “context” that alters the processing of other stimuli. More specifically, the rotation of a planar surface, driven by the subject’s actions, generates specific patterns of transformation within the optic array. When these transformations involve individual conflicting components while preserving their differences, the resulting metastimulus behaves as a higher-order structure that remains invariant over change^58^, leading to the perception of a “new” coherent, unitary object. All the potential deformations that it undergoes to, due to the observer’s action, constitute the set of contingencies that characterize that specific kind of object^59^.

### The role of manipulating sensorimotor contingencies

The majority of cited studies in visual neuroscience and experimental psychology, utilize synthetic stimuli, highly controlled conditions, and established toolboxes to investigate how visual information is extracted and represented in the brain, often overlooking dynamic aspects. To incorporate dynamics and real-time manipulation of stimuli presented to subjects, there is a critical need for tools that facilitate this progress more effectively. Virtual reality represents a promising technology with significant potential for designing sophisticated experiments in behavioral vision science and psychology research more broadly. Specifically, for actively manipulating the visuomotor contingencies required in the present study, we developed a novel methodology and experimental setup that enable the creation of dynamic and conflicting visual stimuli, where the stimulus’ characteristics changes in response to the subject’s movements in an actual closed perception-action loop. Active manipulation of visual cues that define the stimulus can disclose unknown (and more ecological) aspects of perception, resulting from continuous sensorimotor contingencies and their temporal dynamics. This could pave the way to a novel approach to visual psychophysics that complements traditional forced-choice paradigm with target tracking procedures, in order to provide insights into real-time dynamics of perception and action, aligning to and extending the promises/potential of continuous psychophysics^60,61^.

## Supplementary Information

Supplementary Information.

## Data Availability

The data supporting the findings of this study are not publicly available. They can be obtained from the corresponding author upon reasonable request.
