# Supplementary material for: Depth perception changes following adaptation to cue-dependent invariants
Source: Sci Rep. 2025 Dec 30;15:45741. doi: 10.1038/s41598-025-28193-1 (PMC12756225; doi:10.1038/s41598-025-28193-1)
Supplement: Supplementary file 1 — Supplementary Information. [file 41598_2025_28193_MOESM1_ESM.pdf]

Supplementary Information

Depth Perception Changes Following Adaptation to Cue-dependent Invariants

Francesca Peveri[1,*], Federico Barban[1], Andrea Canessa[1], and Silvio P. Sabatini[1]

[1]Department of Informatics, Bioengineering, Robotics and Systems Engineering University of Genoa - via Opera Pia 11a, 16145, Genoa, ITALY
[*]francesca.peveri@edu.unige.it

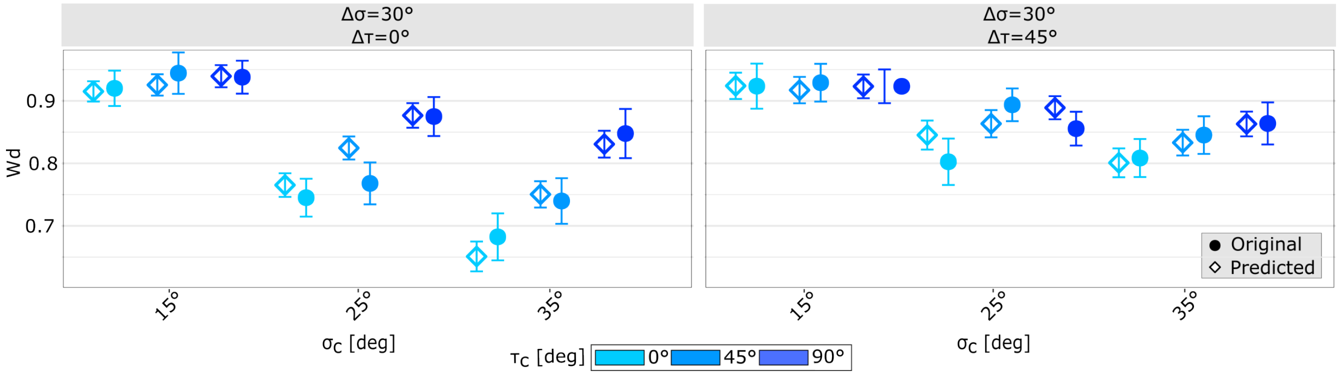

Figure S 1. Original vs. predicted data across experimental conditions. Comparison of participant-averaged (n = 29) original and mixed-model predicted disparity cue weights w_d from the perceptual judgment Task across slant values (σ_c = 15°, 25°, 35°), grouped by tilt values (τ_c = 0°, 45°, 90°) using different fill-in colors. The mean of the original data (circles) and model predictions (diamonds) are shown with 95% confidence intervals (CI, error bars).

Model	$(\Delta\sigma, \Delta\tau)$	AIC	BIC	R_c^2	R_m^2	RMSE	Sigma
glmer	(30,0)	-1365.302	-1308.775	0.388	0.205	0.144	0.123
glmer	(30, 45)	-1839.577	-1782.876	0.286	0.073	0.121	0.107

Table S 1. Model metrics for generalized linear mixed-effects models (GLMM, fitted with `glmer`) investigating the effect of σ_c and τ_c effects on the disparity weights. Metrics include Akaike Information Criterion (AIC), Bayesian Information Criterion (BIC), R_c^2 (explaining fixed and random effects variance), marginal R_m^2 (explaining fixed effects variance), root mean square error (RMSE), and residual standard deviation (Sigma).

Model	Group	$(\Delta\sigma, \Delta\tau)$	AIC	BIC	R_c^2	R_m^2	RMSE	Sigma
lmer	ATD	(30,0)	-157.12	-110.99	0.80	0.28	0.10	0.10
lmer	.	(30, 45)	-192.18	-145.96	0.78	0.25	0.09	0.09
lmer	AD	(30,0)	-162.54	-114.90	0.84	0.15	0.10	0.11
lmer	.	(30, 45)	-203.80	-156.25	0.84	0.11	0.09	0.09
lmer	AT	(30,0)	-208.32	-160.43	0.74	0.32	0.10	0.10
lmer	.	(30, 45)	-282.58	-234.86	0.77	0.1	0.07	0.08

Table S 2. Model metrics for linear mixed-effects models (LMM, fitted with `lmer`) investigating the effect of training on the disparity weights. Metrics include Akaike Information Criterion (AIC), Bayesian Information Criterion (BIC), R_c^2 (explaining fixed and random effects variance), marginal R_m^2 (explaining fixed effects variance) computed with Nagelkerke method[1], root mean square error (RMSE), and residual standard deviation (Sigma).

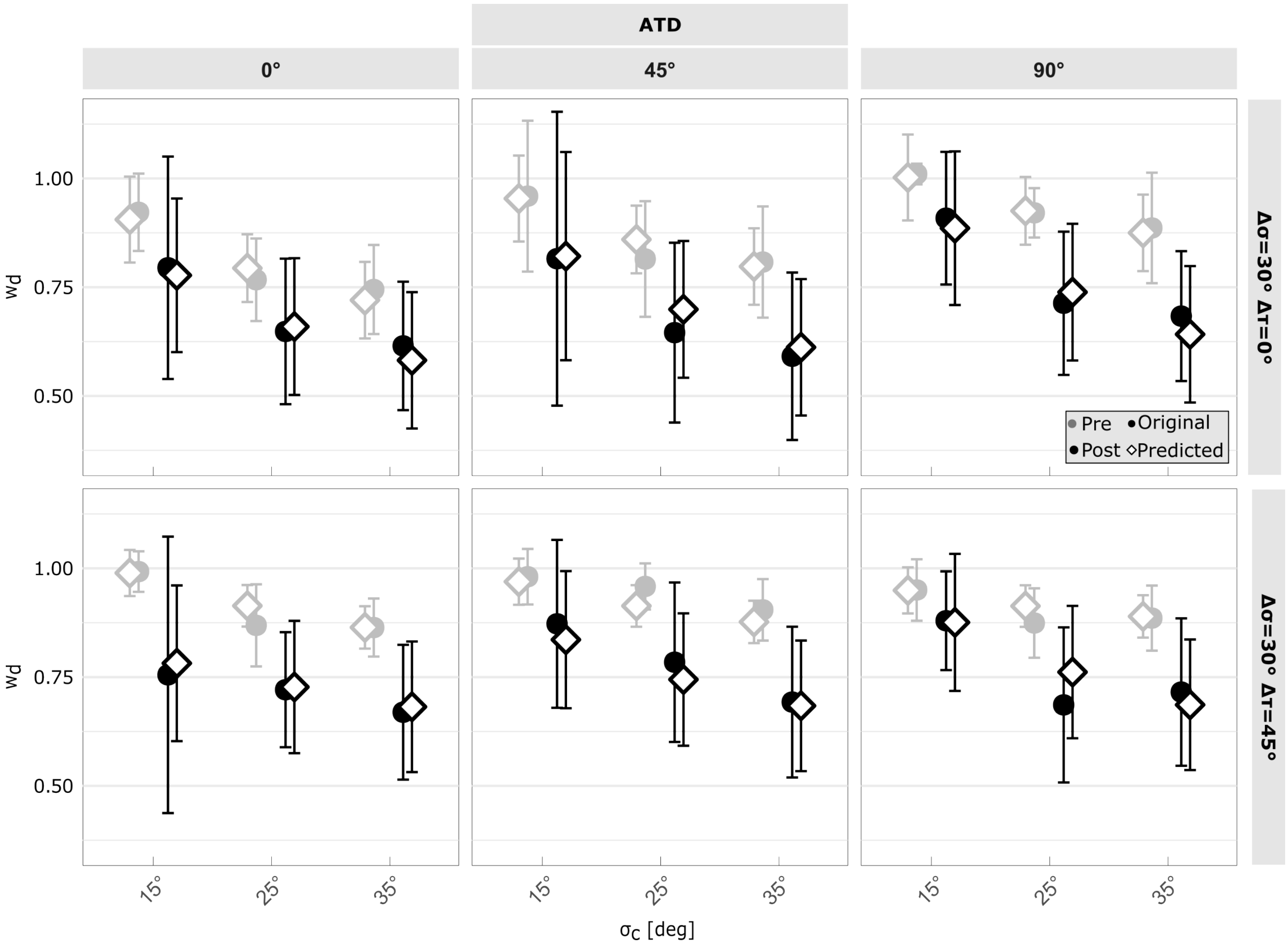

Figure S 2. Original pre- and post-training disparity cue wights vs. pre- and post- predictions for ATD group. Disparity cue weights w_d before (gray circles) and after training (black circles) with mixed-model predictions (white diamonds with matching borders) for the ATD experimental group. The plot shows central slant values (σ_c = 15°, 25°, 35°) on the x-axis, with columns grouping data by tilt (τ_c = 0°, 45°, 90°), and rows representing different discrepancy conditions. The mean (n = 9) of the original data model predictions are shown with 95% confidence intervals (CI, error bars).

References

1. Nakagawa, S., Johnson, P. C. & Schielzeth, H. The coefficient of determination r 2 and intra-class correlation coefficient from generalized linear mixed-effects models revisited and expanded. *J. Royal Soc. Interface* **14**, 20170213 (2017).

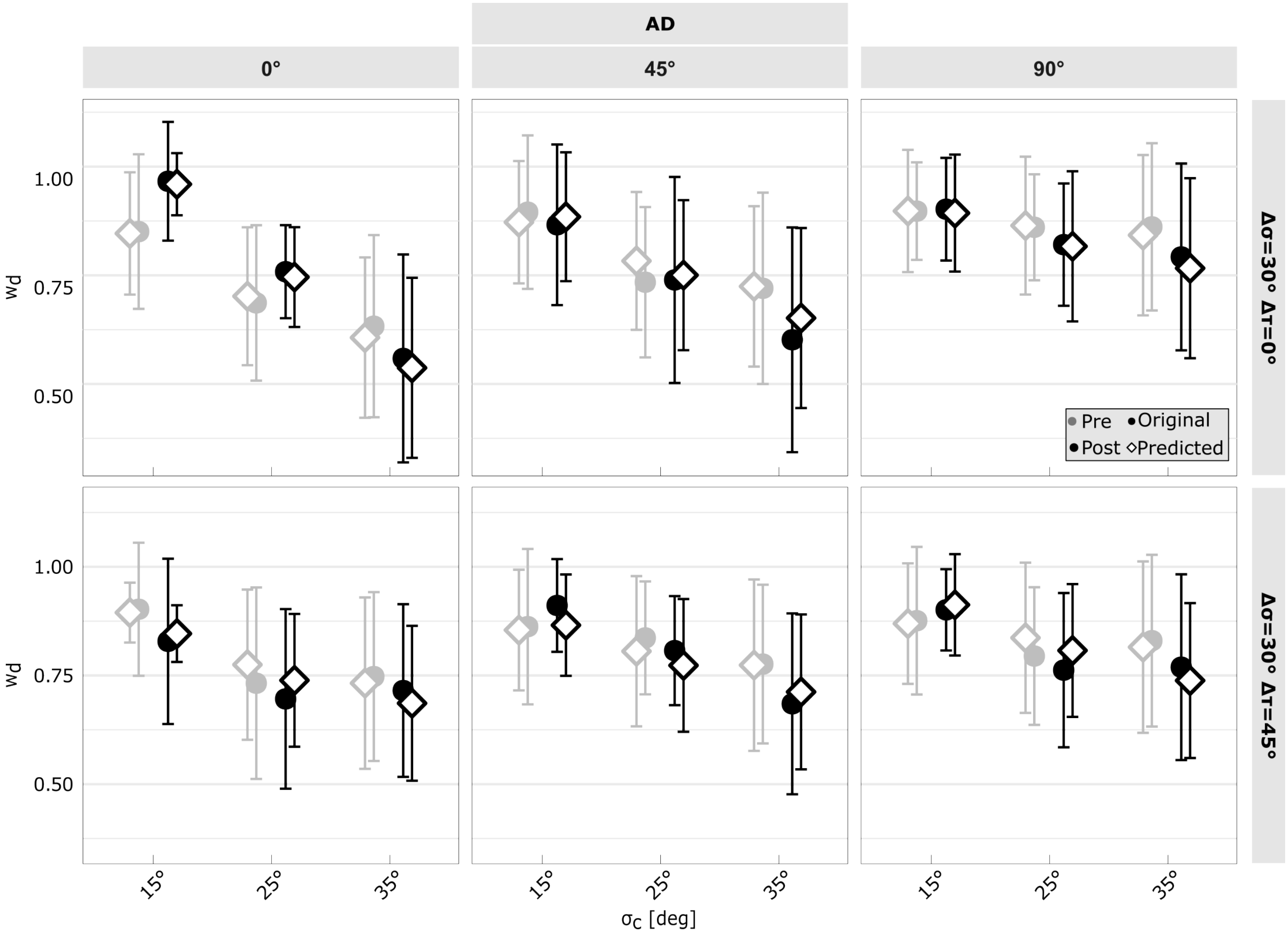

Figure S 3. **Original pre- and post-training disparity cue wights vs. pre- and post- predictions for AD group.** Disparity cue weights w_d before (gray circles) and after training (black circles) with mixed-model predictions (white diamonds with matching borders) for the AD experimental group. The plot shows central slant values (σ_c = 15°, 25°, 35°) on the x-axis, with columns grouping data by tilt (τ_c = 0°, 45°, 90°), and rows representing different discrepancy conditions. The mean (n = 10) of the original data model predictions are shown with 95% confidence intervals (CI, error bars).

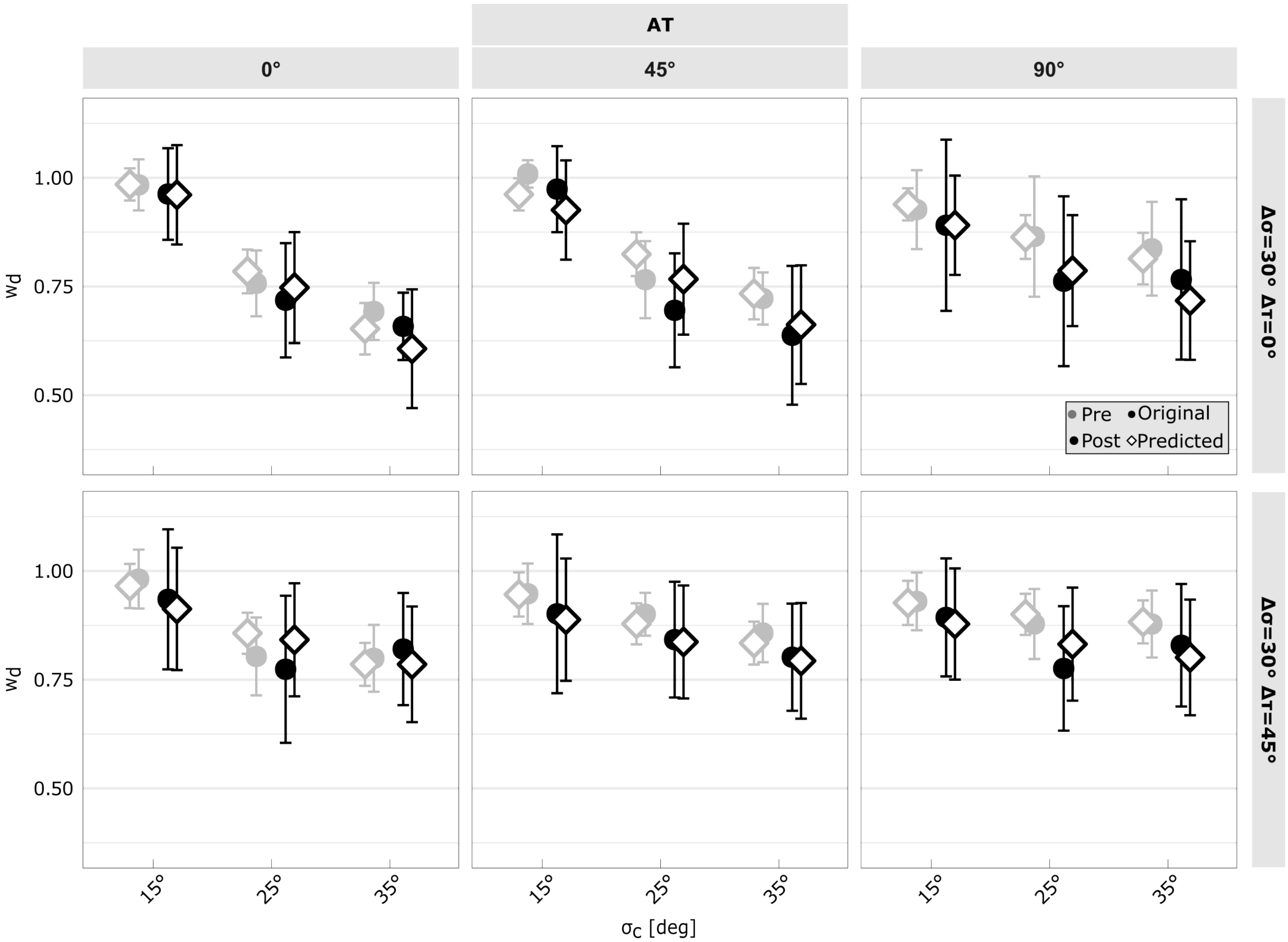

Figure S 4. **Original pre- and post-training disparity cue wights vs. pre- and post- predictions for AT group.** Disparity cue weights w_d before (gray circles) and after training (black circles) with mixed-model predictions (white diamonds with matching borders) for the AT experimental group. The plot shows central slant values (σ_c = 15°, 25°, 35°) on the x-axis, with columns grouping data by tilt (τ_c = 0°, 45°, 90°), and rows representing different discrepancy conditions. The mean (n = 10) of the original data model predictions are shown with 95% confidence intervals (CI, error bars).

Group	σ_c	τ_c	$(\Delta\sigma, \Delta\tau)$	effect	SE	df	t-ratio	p-value
ATD	15	0	(30, 0)	-0.1281	0.0561	38.79	-2.283	0.0280
.	25	0	.	-0.1343	0.0450	16.42	-2.984	0.0086
.	35	0	.	-0.1383	0.0534	32.38	-2.589	0.0143
.	15	45	.	-0.1223	0.0456	17.42	-2.680	0.0156
.	25	45	.	-0.1606	0.0402	10.19	-3.993	0.0025
.	35	45	.	-0.1858	0.0441	15.14	-4.210	0.0007
.	15	90	.	-0.1165	0.0561	38.79	-2.076	0.0445
.	25	90	.	-0.1869	0.0450	16.42	-4.153	0.0007
.	35	90	.	-0.2332	0.0534	32.38	-4.366	0.0001
ATD	15	0	(30, 45)	-0.1930	0.0624	24.48	-3.092	0.0049
.	25	0	.	-0.1866	0.0538	13.38	-3.468	0.0040
.	35	0	.	-0.1824	0.0599	20.84	-3.046	0.0062
.	15	45	.	-0.1333	0.0541	13.69	-2.463	0.0277
.	25	45	.	-0.1692	0.0505	10.17	-3.351	0.0072
.	35	45	.	-0.1928	0.0532	12.70	-3.627	0.0032
.	25	90	.	-0.1517	0.0538	13.31	-2.822	0.0141
.	35	90	.	-0.2032	0.0599	20.81	-3.393	0.0028
AD	35	45	(30, 45)	-0.0615	0.0250	30.90	-2.466	0.0194
.	35	90	.	-0.0769	0.0365	98.07	-2.104	0.0380

Table S 3. Statistical results of LMM post-hoc pairwise comparisons (Tukey's correction) for cue weighting analysis across different time points (pre-/post-training) and parameter combinations (σ_c, τ_c) in the ATD, AD, and AT groups. The table reports the effect of cue weighting, standard error (SE), t-ratio, and *p*-values for each comparison.
